# Squamous Cell Carcinoma Metastatic to the Thyroid Gland Diagnosed by Fine-Needle Aspiration Cytology: A Case Report

**DOI:** 10.7759/cureus.82665

**Published:** 2025-04-21

**Authors:** Xiaoying Wei, Zhe Chen, Lihua Zhang, Cao Ma

**Affiliations:** 1 Department of Pathology, Zhongda Hospital, School of Medicine, Southeast University, Nanjing, CHN

**Keywords:** fine-needle aspiration cytology, immunohistochemical features, metastasis, squamous cell carcinoma, thyroid

## Abstract

Fine-needle aspiration cytology of the thyroid gland has been widely used for the initial diagnosis of suspected malignant thyroid nodules and definitive preoperative diagnosis of recurrent and metastatic thyroid cancer. Metastases to the thyroid most commonly arise from tumors in the lungs, kidneys, breasts, and skin. Metastatic squamous cell carcinoma can also be observed in the thyroid, but it is relatively rare. Here, we report a case of esophageal squamous cell carcinoma metastatic to the thyroid that was diagnosed using fine-needle aspiration cytology, relying on cytomorphological characteristics, immunohistochemical features, and other clinical clues.

## Introduction

The incidence of metastases to the thyroid ranges from 2.7% to 4% [1]. The most common sites of origin for such metastases are the kidney, lung, and breast [2]. The incidence of metastasis of gastrointestinal malignancies to the thyroid is low, and most are colorectal adenocarcinomas [2]. Thyroid metastasis from esophageal tumors is relatively rare, most of which are metastatic squamous cell carcinomas [3]. Only a small number of case series and small sample studies of metastatic thyroid tumors have been published [3,4].

Here, we report a case of esophageal squamous cell carcinoma metastatic to the thyroid that was diagnosed using fine-needle aspiration cytology (FNAC). We also analyzed its cytopathological features and discussed its differential diagnoses. Identifying this entity is important for the accurate diagnosis of thyroid nodules and appropriate clinical management.

## Case presentation

**Figure 1:**
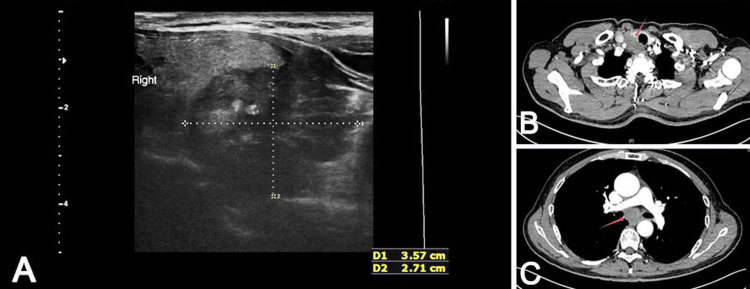
Imaging findings. (A) Neck ultrasound revealed a solid hypoechoic lesion (3.57 × 2.71 × 2.6 cm) in the right thyroid lobe. (B) CT demonstrated a soft tissue mass at the right thoracic inlet involving the right thyroid and trachea. (C) CT showed an irregularly shaped mass in the mid-to-distal esophagus.

A 66-year-old man was admitted to the hospital owing to neck swelling and eating obstruction that worsened progressively over six months. Ultrasonography revealed a solid hypoechoic nodule with an irregular shape, heterogeneous multiple spot-like strong echoes, and an unclear boundary in the right lobe of the thyroid gland (Figure 1A).

**Figure 2:**
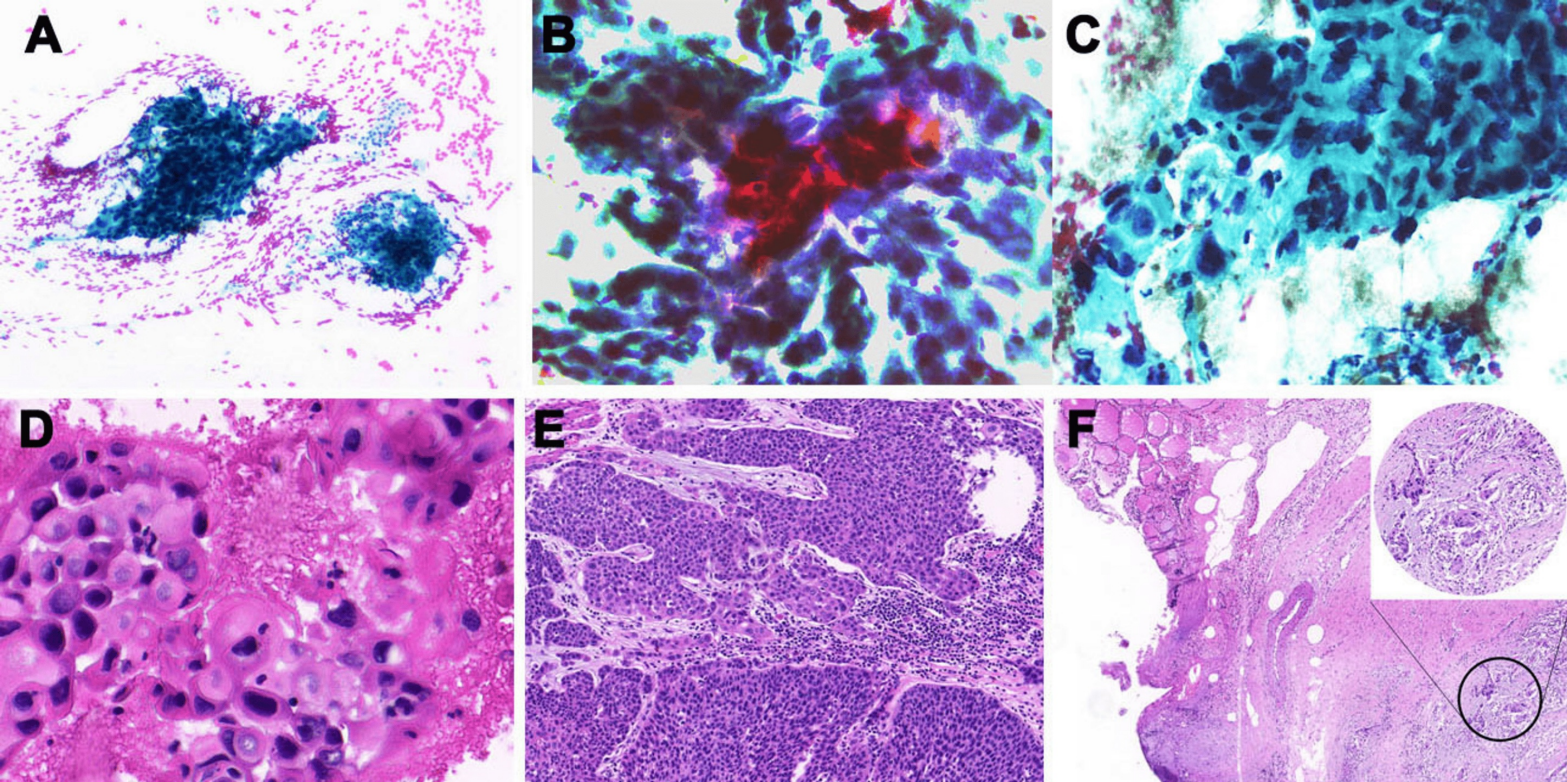
Cytomorphological and histologic features of metastatic squamous cell carcinoma in the thyroid. (A) Clusters of tumor cells are arranged in sheets and nests, with rich and eosinophilic cytoplasm; Papanicolaou stain (Pap; 10×). (B) Tumor cells with moderate to severe atypia and cytoplasmic keratosis are evident in some areas (40×). (C) Tumor cells with a high nucleocytoplasmic ratio, rough chromatin, irregular nuclear membranes, and necrotic debris and inflammatory cells are often observed in the background, Pap (40×). (D) Cell blocks of the thyroid fine-needle aspirate show similar cytological characteristics to the smear; hematoxylin & eosin stain (HE; 40×). (E) A surgical specimen slide from squamous cell carcinoma of the esophagus shows moderately to poorly differentiated tumor cells, HE (10×). (F) The thyroidectomy specimen shows a similar histological appearance to that of the esophageal lesions, HE (5×).

Ultrasound-guided fine-needle aspiration was performed, a smear was prepared, and the residual material was immediately fixed in 95% ethanol to prepare cell blocks. Papanicolaou staining was performed on the smear, and cell block sections were stained with hematoxylin and eosin. The smear showed that the tumor cells were scattered or distributed in sheets and nests, with abundant and eosinophilic cytoplasm. Benign follicular cells arranged in monolayer sheets or honeycomb-like structures were observed in the background (Figure 2A).

**Figure 3:**
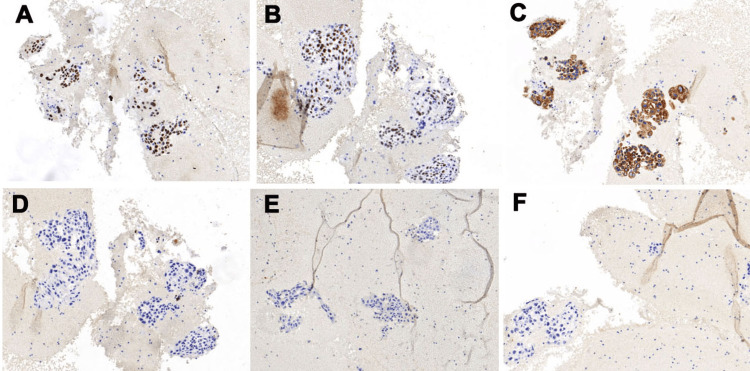
Immunohistochemistry on cell block sections. Staining for (A) p63, (B) p40, (C) CK5/6, (D) TTF1, (E) thyroglobulin, and (F) PAX8 (all at 10×).

The tumor cells varied in size and shape, with moderate-to-severe atypia, and cytoplasmic keratinization was seen in some areas (Figure 2B). The tumor cells also showed unusual nuclear shapes, a high nucleocytoplasmic ratio, hyperchromatic nuclei, clumped chromatin, and irregular nuclear membranes. Necrotic debris and inflammatory cells were present in the background (Figure 2C). The cell blocks showed cytological characteristics like those of the smears (Figure 2D). Immunocytochemistry was performed on the cell blocks using the Dako Omnis automated immunostaining machine (Agilent, Santa Clara, CA) following the steps of EnVision. This revealed that the tumor cells were strongly positive for p40, p63, and CK5/6, and negative for TTF1, thyroglobulin, and PAX8 (Figure 3).

Combined with the morphological characteristics and immunotyping, thyroid squamous cell carcinoma was considered, although FNAC alone could not determine whether this was a primary or secondary tumor.

The patient subsequently underwent simultaneous computed tomography of the chest and upper abdomen owing to a further symptom, i.e., esophageal obstruction. The results revealed irregular thickening of the esophageal wall with luminal narrowing, indicating potential thyroid invasion (Figures 1B, 1C). The patient had a high probability of an esophageal malignant tumor with thyroid metastasis, which was confirmed through close clinical evaluation. In view of the late stage of the tumor, two cycles of neoadjuvant chemotherapy (paclitaxel + cisplatin + sintilimab) were administered before surgery. Thoracoscopic radical resection of the esophageal cancer and right thyroidectomy were performed after chemotherapy. Postoperative pathological findings revealed a moderate-to-poorly differentiated squamous cell carcinoma of the esophagus (Figure 2E). The thyroidectomy specimen showed a histological appearance like that of the esophageal lesions (Figure 2F).

## Discussion

Squamous cell carcinoma of the thyroid (SCCT) is a rare malignant tumor of the thyroid gland that can be divided into primary SCCT (PSCCT) and secondary SCCT (SSCCT) [5]. PSCCT was classified as a subtype of anaplastic thyroid carcinoma in 2022 in the newest World Health Organization classification of thyroid neoplasms [5]. The origin of PSCCT remains controversial. Most scholars believe that squamous cells are derived from embryonic remnants of the ultimobranchial bodies or the thyroglossal duct, while others consider them to originate from metaplasia complicated with chronic thyroid diseases [4,6]. SSCCT is mostly derived from tumor invasion or metastases from adjacent organs, and the common primary sites of SSCCT are the esophagus, larynx, hypopharynx, and lung/trachea [7-13].

For a long time, PSCCT was believed to have a poor prognosis and was less common than SSCCT [7]. However, a retrospective analysis of 17 patients with PSCCT and six patients with SSCCT [4] indicated that the mean and median survival times of patients with PSCCT were longer than those with SSCCT [6]. We speculate that the limited sample size and selection bias may account for this prognostic difference. We conducted a systematic literature search in PubMed from 2014 to 2024 using the following key terms: ("esophagus" OR "esophageal") AND ("metastatic squamous cell carcinoma "OR" secondary squamous cell carcinoma") AND ("thyroid"). This search identified nine relevant English-language publications meeting our inclusion criteria. Table 1 summarizes the clinicopathological characteristics of SSCCT with esophageal origin reported in the literature over the past decade.

**Table 1:** Clinicopathological characteristics of esophageal-derived metastatic squamous cell carcinoma of the thyroid: a 10-year retrospective analysis (2014-2024). NA: no data available. Palliative operation includes tracheotomy and biopsy (without radical resection or radical operation).

Reference	Research methodology	Number of cases	Age/range	Site of primary lesion	Treatment	Survival (months)
Chen et al. (2014) [11]	Case report	1	61	Esophagus	Palliative operation	11
Ye-huan et al. (2015) [12]	Case report	2	50, 54	Esophagus	Palliative operation	15, 7
Ching et al. (2018) [9]	Case report	1	44	Esophagus	Palliative operation	Survival during follow-up
Zhang et al. (2020) [10]	Case report	1	69	Esophagus	Radical operation	Survival during follow-up
Liu et al. (2021) [4]	Retrospective study	6	45-67	Esophagus = 2, larynx = 1, hypopharynx = 1, lung/trachea = 2	Palliative operation = 3, radical operation = 3	Median survival: 3
Zhao, et al. (2022) [7]	Case report	1	69	Esophagus	Palliative operation	Survival during follow-up
Saini et al. (2023) [3]	Retrospective study	9	29-69	Esophagus	NA (histological biopsies were performed in at least six cases)	NA
Zhang et al. (2024) [14]	Retrospective study	6	51-72	Esophagus = 3, larynx = 3	Palliative operation	NA
Shaikh et al. (2024) [15]	Case report	1	30	Esophagus	Palliative operation	Survival during follow-up
Present case	Case report	1	66	Esophagus	Radical operation	Survival during follow-up

SSCCT patients were generally older at disease onset, with a mean age of 56.9 years (range = 29-72 years). Patients usually had an advanced-stage malignant tumor, most of whom only received palliative surgery, resulting in a poor overall prognosis. However, the clinical information of some patients was incomplete, and more detailed prognostic information should rely on a larger sample of case studies.

Cytology alone cannot accurately distinguish between PSCCT and SSCCT; immunohistochemistry may provide auxiliary information. Thyroid transcription factor 1 (TTF1) is considered a useful immunohistochemical marker for diagnosing thyroid and lung tumors, though its role in PSCCT is minimal [6]. Approximately 17% of PSCCT are TTF1 positive [12], indicating that TTF1 is almost indistinguishable from PSCCT and SSCCT. Pax8 is expressed in approximately 80% of mesenchymal thyroid cancer cases. The diagnosis of PSCCT is better supported when PAX8 expression is positive, whereas negative expression tends to indicate metastatic SCC from other sites [13,14].

Patients with metastatic SCCT typically present at advanced disease stages, necessitating multimodal therapeutic approaches. Effective management requires concurrent treatment of both metastatic lesions and primary foci, incorporating chemotherapy, radiotherapy, and surgical intervention where appropriate [15]. Post-treatment surveillance is essential, with close follow-up enabling early detection and prompt intervention for disease recurrence or new metastases.

## Conclusions

For thyroid cancers with uncommon cytological morphologies, the possibility of metastatic thyroid cancer should be considered during diagnosis. The diagnosis of SSCCT based solely on cytomorphological features presents significant diagnostic challenges. Therefore, comprehensive integration of clinical correlation is imperative for accurate classification. Accurate diagnosis and appropriate treatment may help improve the patient's quality of life and prolong survival.
